# Peri‐Implant Health and Perfusion Parameters in Patients After Microvascular Jaw Reconstruction: A Clinical Cohort Study

**DOI:** 10.1111/cid.70012

**Published:** 2025-02-12

**Authors:** Marie Sophie Katz, Mark Ooms, Marius Heitzer, Anna Bock, Nils Vohl, Kristian Kniha, Frank Hölzle, Ali Modabber

**Affiliations:** ^1^ Department of Oral and Maxillofacial Surgery University Hospital RWTH Aachen Aachen Germany

**Keywords:** laser doppler flowmetry, microvascular flap, perfusion, peri‐implant mucositis, peri‐implantitis, tissue spectrophotometry

## Abstract

**Introduction:**

The aim of this study was to evaluate perfusion parameters and clinical features of healthy implants and implants affected by peri‐implant disease in patients who had undergone microvascular jaw reconstruction.

**Methods:**

A total of 25 patients with 92 implants placed in microvascular transplants were included. Of these, 68 implants showed healthy peri‐implant tissue, 12 were affected by peri‐implant mucositis, and 12 were diagnosed with peri‐implantitis. Peri‐implant perfusion was measured mesially and distally at the implant shoulder using laser Doppler flowmetry and tissue spectrophotometry (LDF‐TS), followed by a clinical evaluation, including measurement of probing depths, bleeding on probing (BOP), plaque index, biotype, type of implant, the restoration and the presence of keratinized tissue. Perfusion parameters were compared between the healthy implants and the implants with peri‐implant disease based on the conventional BOP–based diagnosis of peri‐implantitis, and the associations between the perfusion values and clinical measurements were analyzed. Optimal cut‐off values for predicting peri‐implantitis were calculated with receiver operating characteristics.

**Results:**

The mean relative amount of hemoglobin and mean blood flow were significantly different between healthy implants and implants with peri‐implant mucositis and peri‐implantitis (*p* = 0.003 and *p* = 0.002, respectively). However, there are interindividual differences that appear to influence blood flow values as well. When a linear mixed regression model was applied, including the patient as a random variable, the difference in blood flow was no longer statistically significant (*p* = 0.400). Still, the optimal cut‐off value of mean blood flow for predicting peri‐implantitis was determined to be > 46.5 AU (AUC = 0.788; *p* < 0.001; CI = 0.695–0.881; sensitivity = 1.00, specificity = 0.60).

**Conclusion:**

Implants in microvascular flaps are particularly vulnerable to peri‐implant disease. Risk factors are the lack of keratinized peri‐implant tissue, fixed restorations, bone‐level implants, and high plaque levels.

As a noninvasive and objective method, LDF‐TS can contribute to risk assessment by evaluating perfusion parameters and help detect the early onset of peri‐implant disease.

## 1 Introduction

Dental rehabilitation is the last step of a long journey for patients who have experienced oral cancer, trauma, osteomyelitis, or other conditions that require resection of parts of the mandible or maxilla and later microvascular reconstruction with, for example, a free fibula flap (FFF) or a deep circumflex iliac artery (DCIA) transplant. Dental implants help to retain fixed or removable dentures in altered bone and soft tissue surroundings and therefore have an important role in improving and restoring patients' quality of life [1, 2].

Several parameters have been identified that influence peri‐implant health: implant‐related factors, such as the amount of keratinized tissue, the type or material of the implant, the cementation, and type of restoration, as well as patient‐related factors, such as smoking, diabetes, radiation, and oral hygiene [3, 4, 5, 6, 7, 8].

Scarring and uneven distribution of soft tissue in patients who have undergone microvascular jaw reconstruction, as well as resorption of the transplanted bone, and difficulties with oral hygiene can make peri‐implant health a challenge [9, 10, 11]. Previous studies have shown that implants in microvascular reconstructed areas are more vulnerable to peri‐implant mucositis and peri‐implantitis compared with implants in native bone [1, 10, 12].

The diagnosis of peri‐implant disease is mainly based on bleeding on probing (BOP) because implants with peri‐implant mucositis and peri‐implantitis show typical inflammation signs, such as hyperemia, redness, and suppuration around the implant shoulder [8, 13, 14, 15]. However, as implants do not have a natural periodontium, BOP has been highly associated particularly with pressure on the gingival probe, and especially around implants, the results are often false positive, as Dukka et al. showed [16].

On the other hand, a recent study by Ramanauskaite et al. showed that progressive bone loss at implant sites is not always linked with BOP [17].

A few attempts have been made to visualize vascularity and perfusion around dental implants by ultrasound, but although ultrasound can show some differences between healthy and inflamed peri‐implant mucosa concerning velocity and echo intensity, it lacks information on the quantification of hyperemia and blood flow and on vessel constriction [18, 19].

Laser Doppler flowmetry and tissue spectrophotometry (LDF‐TS) is a commonly used tool to monitor microvascular transplants [20, 21, 22, 23, 24]. By using a probe with a laser and a white light it can detect not only blood flow but also the relative amount of hemoglobin rHb (AU) and oxygen saturation SO2 (%), which can lead to a deeper understanding of the underlying tissue vitality. It has been successfully used in studies evaluating mucosa perfusion and gingival health in natural teeth and to measure perfusion differences of the mucosa around implants placed in different bone grafts [25, 26, 27]. It is therefore a promising diagnostic tool to further understand peri‐implant processes that affect peri‐implant health and could thus be a useful addition to clinical examination beside BOP measurements and visual inspection.

The primary aim of this study was to evaluate if peri‐implant perfusion around implants placed in microvascular bone transplants is different between healthy implants and implants affected by various levels of peri‐implant disease, based on the conventional BOP‐based diagnosis of peri‐implantitis.

Furthermore, a cut‐off perfusion value corresponding to the clinical diagnosis of peri‐implantitis should be identified.

In addition to the use of LDF‐TS, the secondary aim of this study was to analyze risk factors in implants from a cohort that underwent microvascular reconstruction and implant placement. The implants were categorized based on implant type, prosthetic approach, and demographic factors.

## 2 Materials and Methods

### 2.1 Study Design

This study was approved by the local clinical research ethics committee (Decision Number 23–108) and registered at the German Clinical Trials Register (File Number DRKS00034432).

All procedures performed in this study were in accordance with the ethical standards of the 1964 Helsinki Declaration. Informed consent was obtained from all individual participants included in the study.

#### 2.1.1 Eligibility Criteria

All participants in this study were recruited during their regular implant recalls in our clinic. To be included, patients had to be at least 18 years of age and to have had at least one dental implant inserted into a microvascular bone transplant (FFF or DCIA). The exclusion criteria were pregnancy, smoking less than two hours before the examination, and acute signs of abscess or intraoral swelling.

#### 2.1.2 Sample Size Calculation

The literature on clinical trials of peri‐implant health in patients who had undergone microvascular reconstruction and on peri‐implant perfusion methods was reviewed to calculate a suitable sample size range [1, 12, 18, 19].

The required sample size was derived based on a study by Barootchi et al. [18], who used color flow ultrasonography to evaluate peri‐implant tissue perfusion, and on a study by Galarraga‐Vinueza [19], who evaluated echo‐intensity characterization at implant sites with and without peri‐implantitis. Blood flow differences between healthy implants and peri‐implant disease were defined as the primary outcome to calculate the sample size.

The statistical program G* Power Version 3.1.9.6 (Heinrich‐Heine‐Universität, Düsseldorf, Germany) was used, with an alpha value of 0.05, an effect size of 0.80, and a statistical power of 80%. Based on these parameters, a total sample size of 42 implants (21 with and 21 without peri‐implant disease) was determined to reject the null hypothesis concerning blood flow differences with 80% power and a 95% confidence interval.

### 2.2 Perfusion Measurement

The examinations always started with the LDF‐TS perfusion measurements to ensure that the peri‐implant tissue was not manipulated by the clinical examination before.

**FIGURE 1:**
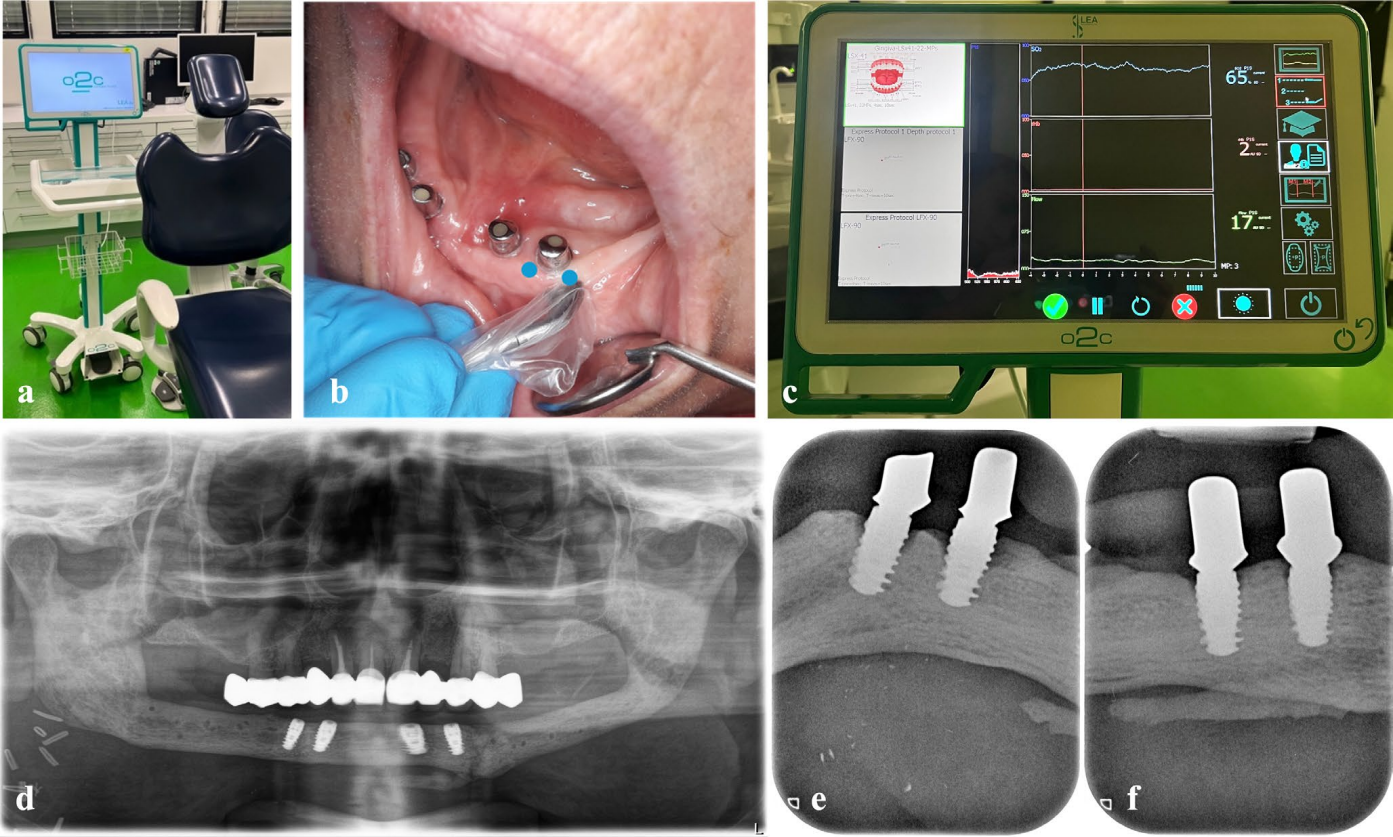
“Oxygen to see” (O2C) device (a), its gingival probe (b) and measurement setting (c), the initial situation directly after implantation of the FFF (d), and the later onset of peri‐implantitis in two of the four implants (e and f).

An “oxygen to see” (O2C) device (LEA‐Medizintechnik, Gießen, Germany) was used to measure oxygen saturation SO_2_ (%), the relative amount of hemoglobin rHb (Arbitrary Units [AU]), and blood flow (AU) with a laser and a white light. For intraoral use, a specialized gingiva probe sized 5 × 2 mm with a measurement depth of 1 mm was used (LSX‐41 gingival probe, LEA‐Medizintechnik, Gießen, Germany) (Figure 1).

The patient laid on the dental chair in a comfortable horizontal position, and the dental chair light was switched off to avoid measurement alterations. The measurement was performed mesially and distally on the buccal side of the implant, which means the sensor was 2 mm away from the implant shoulder to ensure standardized and reproducible measurements, and to prevent the implant shoulder from shimmering through the gingiva. Afterwards, the mean perfusion values were calculated. Each measurement took 10 s per point and was conducted by the same dentist. If the examiner saw any disturbances or unexpected movements, the measurement was immediately repeated.

### 2.3 Clinical Examination and Classification

After the perfusion measurement, the implants were examined clinically. This included measuring the pocket depths and plaque index and identifying the biotype and presence of keratinized gingiva, clinical inflammation signs, and BOP. The clinical classification of implant health and disease was based on the review by Renvert et al. [14]. Peri‐implant health was defined as the absence of peri‐implant signs of soft tissue inflammation (redness, swelling, and profuse BOP) and the absence of further additional bone loss following initial healing. Peri‐implant mucositis was diagnosed when there were signs of peri‐implant inflammation, such as redness, swelling, a line, or drop of blood within 30 s following probing but no additional bone loss. If implants showed peri‐implant inflammation, there was evidence of radiographic bone loss ≥ 3 mm, and a probing depth ≥ 6 mm, they were assigned to the peri‐implantitis group.

Since BOP can yield false negatives, we classified implants that exhibited visible signs of inflammation, such as redness or swelling of the gingiva, but no BOP, as affected by peri‐implant mucositis, provided there was no additional bone loss. If there was additional bone loss, these implants were classified as affected by peri‐implantitis. On the other hand, as suggested by Renvert et al. [14], we classified implants as healthy if there were no signs of inflammation, except for a small dot of BOP.

In addition, the implant type and material, type of restoration, cementation of abutments, and type of peri‐implant tissue (e.g., oral mucosa or cutaneous flap) were documented. The patients' medical record was checked for the reason for the microvascular reconstruction, history of radiation, former implant loss in the reconstructed area, and avascular augmentation procedures that followed microvascular reconstruction before implantation.

Subsequently, the perfusion values, the data from the clinical examination, and background information were matched and statistically analyzed.

### 2.4 Statistical Analysis

For the categorical data (peri‐implant disease, sex, microvascular transplant type, reason for reconstruction, smoking habits, radiation, history of implant loss, implant location, type, material, gingival biotype, presence of keratinized gingiva or cutaneous transplant, former avascular augmentation, plaque index, type of restoration, and restoration mode), the differences between groups were analyzed using the Freeman–Halton test. The continuous data (age, mean time since insertion, mean oxygen saturation, mean relative amount of hemoglobin, and mean blood flow) were evaluated using the Mann–Whitney test or the Kruskal–Wallis test, as they lacked a Gaussian distribution according to the Shapiro–Wilk test. A linear mixed regression model was used to test for differences in blood flow (dependent variable), with the patient as the random independent variable and the type of microvascular transplant, implant material, keratinized gingiva, and implant type as fixed independent variables, as these were considered to potentially influence local blood flow.

The receiver operating characteristics (ROC) were calculated, and the Youden Index was used to determine the theoretically optimal cut‐off value of mean blood flow for predicting peri‐implantitis. P‐values < 0.05 were considered to be significant.

## 3 Results

A total of 25 patients (12 males and 13 females) with 92 implants were included. Of these, 19 patients had undergone an FFF, and 6 patients had a DCIA transplant. The mean time since implant insertion was 47.3 months (SD ± 35.9). Five patients had experienced a former implant loss (6 implants in total) in the same microvascular transplant that was measured (total survival rate of this cohort: 94%) (Table 1).

**TABLE 1:** Patient cohort and characteristics.

	Healthy	Peri‐implant mucositis	Peri‐implantitis	Total	*p*
Sex					
Male	8	2	2	12	0.293
Female	12	1	0	13	
Mean age (years)					
	57.9 (SD ± 14.2)	52.7 (SD ± 15.5)	61.0 (SD ± 14.1)	57.5 (SD ± 13.8)	0.792
Type of microvascular transplant					
FFF	15	3	1	19	0.519
DCIA	5	0	1	6	
Reason for reconstruction					
Malignancy	9	2	2	13	0.486
Benign disease	11	1	0	12	
Smoking habits					
Yes	3	0	0	3	1.000
No	17	3	2	22	
Radiation					
Yes	6	2	1	9	0.464
No	14	1	1	16	
History of implant loss in the microvascular transplant					
Yes	4	1	0	5	0.708
No	16	2	2	20	

Abbreviations: FFF = free fibula flap, DCIA = deep circumflex iliac artery transplant.

A total of 68 implants were diagnosed as healthy, 12 showed peri‐implant mucositis, and 12 were affected by peri‐implantitis (Table 2).

**TABLE 2:** Characteristics of the implants inserted into microvascular transplants.

	Healthy	Peri‐implant mucositis	Peri‐implantitis	Total	*P*
Location					
Maxilla	21	2	1	24	0.223
Mandible	47	10	11	68	
Mean time since insertion of the implants (months)					
	46.9 (SD ± 35.1)	31.8 (SD ± 22.5)	65.3 (SD ± 45.2)	47.3 (SD ± 35.9)	0.120
Implant type					
Bone level	31	10	9	50	**0.023** *
Tissue level	37	2	3	42	
Implant material					
Titanium	56	9	6	71	0.058
Zirconia	12	3	6	21	
Gingival biotype					
Thick	62	12	12	86	0.485
Thin	6	0	0	6	
Keratinized gingiva					
Yes	21	1	0	22	**0.024** *
No	47	11	12	70	
Cutaneous transplant next to the implant					
Yes	33	9	8	50	0.177
No	35	3	4	42	
Former avascular augmentation (on top of the microvascular transplant)					
Yes	18	7	2	27	0.065
No	50	5	10	65	
Bleeding on probing (BOP)					
Yes	28	3	8	39	0.113
No	40	9	4	53	
Mean pocket depths (PD) in mm					
	2.37 (SD ± 0.88)	2.81 (SD ± 0.79)	5.14 (SD ± 1.18)	2.79 (SD ± 1.30)	**< 0.001** *
Plaque index					
0	14	0	1	15	**0.041** *
1	24	9	5	38	
2	20	2	1	23	
3	10	1	5	16	
Type of restoration					
Removable	43	5	1	49	**< 0.001** *
Fixed	25	7	11	43	
Restoration mode					
Screw‐retained	43	5	6	54	0.304
Cemented	25	7	6	38	

*Note:* *means the values were statistically significant.

**FIGURE 2:**
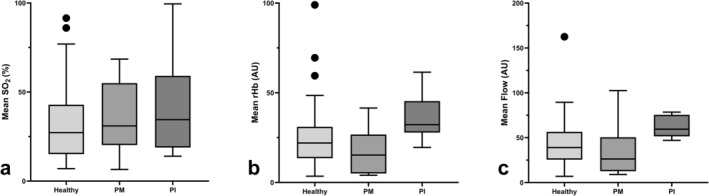
Mean oxygen saturation, relative amount of hemoglobin, and blood flow values compared between the healthy implants and the implants affected by peri‐implant mucositis (PM) or peri‐implantitis (PI). The bars show the median values (SO_2_, rHb, and blood flow), the top and bottom of the boxes show the lower and upper quartile, and the whiskers show lower and upper extreme, excluding outliers. (a) SO_2_ did not differ between the three groups (*p* = 0.382), but (b) rHb and (c) blood flow were significantly different in the implants with peri‐implantitis than in the healthy implants (*p* = 0.003 for rHb and *p* = 0.002 for blood flow).

Comparing the peri‐implant perfusion between the healthy implants with the implants with peri‐implant mucositis and peri‐implantitis, SO_2_ did not differ significantly (*p* = 0.382), but rHb and blood flow were significantly different between the healthy implants and the implants with peri‐implantitis (*p* = 0.003 for rHb and *p* = 0.002 for blood flow; Figure 2). However, there was no significant difference between the healthy implants and the implants with peri‐implant mucositis concerning SO_2_, rHb, and blood flow (*p* = 0.469 for SO_2_, *p* = 0.129 for rHb, and *p* = 0.177 for blood flow).

**FIGURE 3:**
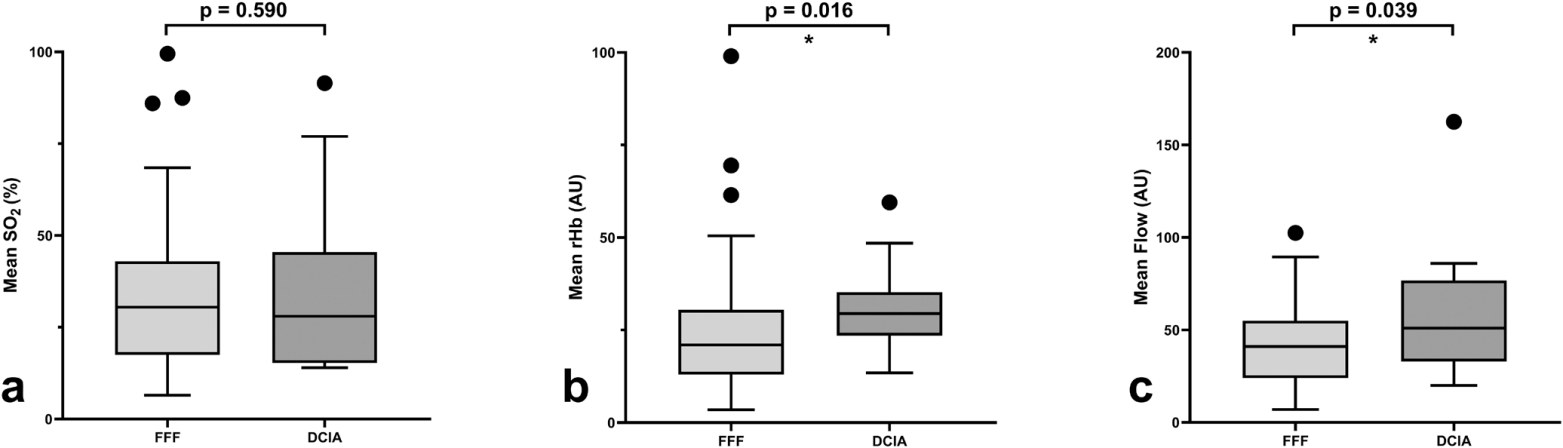
Mean oxygen saturation, relative amount of hemoglobin, and blood flow values compared between implants with an FFF and a DCIA transplant. The bars show the median values (SO_2_, rHb, and blood flow), and the top and bottom of the boxes show the lower and upper quartile, and the whiskers show lower and upper extreme, excluding outlier. (a) SO_2_ did not differ between the two transplant types (*p* = 0.590), but (b) rHb and (c) blood flow were significantly higher in the patients with a DCIA transplant than in those with an FFF (*p* = 0.016 for rHb and *p* = 0.039 for blood flow).

**FIGURE 4:**
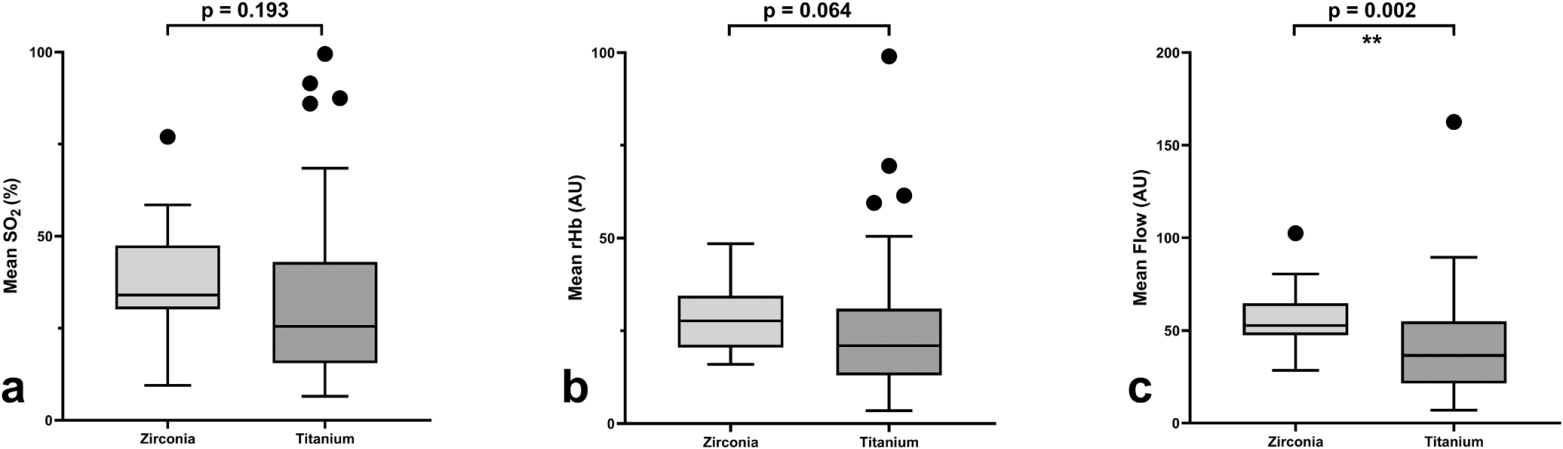
Mean oxygen saturation, relative amount of hemoglobin, and blood flow values compared between the zirconia implants and titanium implants. The bars show the *median* values (SO_2_, rHb, and blood flow), and *the top and bottom of the boxes show the lower and upper quartile, and the whiskers show lower and upper extreme, excluding outliers*. (a) SO_2_ did not differ between the zirconia and titanium implants (*p* = 0.193), nor did (b) rHb (*p* = 0.064), but (c) blood flow was significantly higher around the zirconia implants (*p* = 0.002).

A subanalysis was conducted to assess the influence of factors such as the presence of keratinized gingiva, type of transplant, implant material, type of restoration, and smoking on perfusion parameters, independent of peri‐implant health status: The presence of keratinized gingiva around the implants did not have a significant influence on rHb (*p* = 0.250), or blood flow (*p* = 0.927), but SO_2_ was significantly higher in free gingiva around the implants (*p* = 0.002). While the rHb and blood flow were significantly higher in the patients with a DCIA transplant compared with those with an FFF (*p* = 0.016 for rHb, and *p* = 0.039 for blood flow), SO_2_ did not differ between the two microvascular transplant types (*p* = 0.590) (Figure 3). The implant material had a significant influence on the blood flow values (*p* = 0.002) as the zirconia implants showed higher blood flow values, but SO_2_ and rHb did not differ significantly (*p* = 0.193 for SO_2_ and *p* = 0.064 for rHb) (Figure 4).

Furthermore, rHb (*p* = 0.007) and blood flow (*p* < 0.001) were also significantly higher in the patients with fixed restorations compared with those with removable dentures (*p* < 0.001), but SO_2_ (*p* = 0.198) did not differ.

The implants of smokers showed significantly lower SO_2_ values (*p* = 0.026) and lower rHb values (*p* = 0.047) than implants of the non‐smokers, while blood flow did not differ significantly (*p* = 0.164).

On adjustment for the type of microvascular transplant, implant material, keratinized gingiva, and implant type using a linear mixed regression model, the difference in flow between the healthy implants and the implants affected by peri‐implantitis did not persist (*p* = 0.400).

**FIGURE 5:**
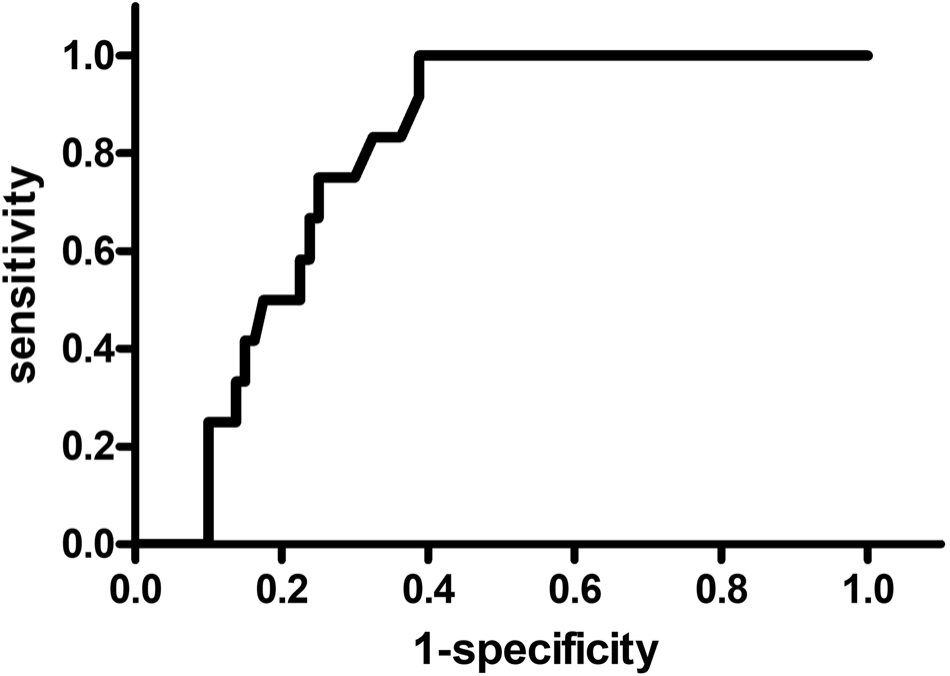
ROC analysis to determine the optimal cut‐off value of mean blood flow for predicting peri‐implantitis. The line corresponds to the mean flow threshold values along with the associated sensitivity and specificity. The optimal cut‐off value of mean blood flow for predicting peri‐implantitis was determined to be > 46.5 AU with 100% sensitivity and 60% specificity (AUC = 0.788; *p* < 0.001; CI = 0.695–0.881).

Performing a ROC analysis, the optimal cut‐off value of mean blood flow for predicting peri‐implantitis was determined to be > 46.5 AU with 100% sensitivity and 60% specificity (AUC = 0.788; *p* < 0.001; CI 0.695–0.881) (Figure 5).

Looking at patient‐related parameters, sex, age, type of microvascular transplant, reason for reconstruction, smoking habits, history of radiation, or former implant loss did not differ between the patients with healthy implants and those who had implants affected by peri‐implant mucositis or peri‐implantitis (*p* = 0.293, *p* = 0.792, *p* = 0.519, *p* = 0.486, *p* = 1.000, *p* = 0.464, and *p* = 0.708, respectively).

Concerning implant‐related factors, a significant difference was found between the healthy implants and those affected by peri‐implant mucositis and peri‐implantitis concerning implant type in favor of bone‐level implants being significantly more vulnerable to peri‐implant disease (*p* = 0.023). Between healthy implants and the implants with peri‐implant mucositis and peri‐implantitis was a significant difference in the presence of keratinized gingiva (*p* = 0.024), in plaque index (*p* = 0.041), and mean pocket depths (*p* < 0.001), as well as in the restoration type (*p* < 0.001). The incidence of BOP was not significantly different between the three groups (*p* = 0.113).

The location of the implants, mean time since insertion, implant material, gingival biotype, presence of a cutaneous transplant, former avascular augmentation, and restoration mode did not differ significantly between the implants with and without peri‐implant mucositis and peri‐implantitis (*p* = 0.223, *p* = 0.120, *p* = 0.058, *p* = 0.485, *p* = 0.177, *p* = 0.065, and *p* = 0.304, respectively).

## 4 Discussion

Patients who have undergone microvascular jaw reconstruction are generally more vulnerable to peri‐implant disease, as shown in previous studies [10, 11, 12, 28]. In this study's cohort, 5 patients had experienced a former implant loss (6 implants in total) in the same microvascular transplant that was measured. This gives an overall survival rate of 94% over a mean insertion time of 47.3 months (SD ± 35.9), which is a lower survival rate compared with implants in native bone [29], but still a favorable outcome overall. The rates of peri‐implant mucositis (13%) and peri‐implantitis (13%) in the 92 implants measured were higher than described for patients with implants in native bone or avascular transplants [30, 31].

As this patient cohort seems to be particularly sensitive to peri‐implant disease compared with patients with implants in native bone with unscarred gingiva, it is even more important to identify peri‐implant disease before it results in implant loss. Perfusion measurement by LDF‐TS could be a useful tool for enhancing diagnostics, especially as bone loss around implants is not always associated with BOP [17].

This study showed that rHb and blood flow were significantly higher in the implants with peri‐implantitis than in the healthy implants. This finding could be explained by vessel alterations that cause congestion, which are present in patients with peri‐implantitis, and hence lead to a higher rHb value, while higher blood flow, accompanied by redness, swelling, and warming, is typical for any kind of inflammation. This is in line with a study by Yamamoto et al. [32], who induced peri‐implantitis in dogs and measured higher blood flow with laser Doppler flowmetry. As they used laser Doppler flowmetry without tissue spectrophotometry, their study did not include rHb values [32]. Moreover, in a study comparing perfusion at the papillae of healthy gingiva to those affected by gingivitis with LDF‐TS, higher rHb and blood flow values at the papilla were found [26].

Age, sex, and microvascular transplant type did not play a significant role when comparing clinically healthy with affected implants. However, the patients with a DCIA transplant showed higher rHb and blood flow values than those with an FFF. This might be due to a greater resorption rate, since an FFF has a higher share of cortical bone. Still, there was no significant difference in the distribution of peri‐implant mucositis or peri‐implantitis. A study by Hocková et al., who compared DCIA transplants with FFF, also found similar rates of implant survival between the two types of transplants [33]. In a study comparing peri‐implant perfusion of avascular and microvascular bone transplants, no differences were detectable between native bone, avascular grafts, or microvascular transplants [27].

Smoking habits and a history of radiation did not differ significantly between the patients with and without peri‐implant disease. This could be due to the small number of smokers (only 3 patients with a total of 10 implants), since other studies have shown that smokers were affected more often by peri‐implantitis [34, 35]. Furthermore, it must be noted that the microvascular transplants were not radiated, since all patients were reconstructed with bone transplants after resection and radiation. However, it is known that these patients experience xerostomia and are generally more vulnerable to peri‐implant disease [36].

Concerning the implant characteristics, the bone‐level implants showed higher rates of peri‐implant mucositis and peri‐implantitis. This may be because that patients with microvascular bone transplants often exhibit difficult soft tissue surroundings, and an implant–abutment connection above the marginal bone level might therefore be beneficial in bridging this larger distance. This finding is in line with a study by Canullo et al. [37], although platform switching seems to be more important than the implant level itself [38]. Apaza‐Bedoya et al. also found a correlation between peri‐implantitis and abutment transmucosal height and hygiene difficulty [39].

All zirconia implants measured in this cohort were tissue‐level implants. Peri‐implant disease was present in 42.8% of the zirconia implants (14.2% with peri‐implant mucositis and 28.5% with peri‐implantitis) and in 21.1% of the titanium implants (12.7% with peri‐implant mucositis and 8.4% with peri‐implantitis), but this finding was not significantly different (*p* = 0.058). The zirconia implants also showed higher blood flow values than the titanium implants. Although rates for peri‐implant disease were not significant, as 42.8% of zirconia implants in this cohort showed peri‐implant disease, there seems to be a strong association with perfusion values. In line with that, the fixed restorations not only showed higher rates of peri‐implant disease but also higher rHb and blood flow values. An explanation could be that in this study, all the zirconia implants had cemented restorations, while the titanium implants were mostly screw‐retained restored (76.1%), and only 23.9% were restored by cementation. Although cementation could not be identified as a significant risk factor in this cohort, previous studies have found that cementation can lead to peri‐implant disease [8, 39].

Another factor might be the kind of restoration. The fixed restorations showed significantly more peri‐implant disease in the patients with microvascular transplants than the removable dentures did. This could be explained by difficulties in cleaning these restorations, as most of the patients had altered soft tissue around the implants, scars, and often a restricted mouth opening. Likewise, Lombardo et al. found that poor oral hygiene practices and compliance in patients who underwent reconstruction with an FFF were also associated with peri‐implant disease [12].

The patients with peri‐implant mucositis and peri‐implantitis also showed significantly less often keratinized gingiva around the implants. This issue should be addressed by vestibuloplasty and the use of free gingival grafts. Numerous previous studies have also found that this is an important risk factor in implants in native bone [40, 41, 42] as well as in implants based on microvascular transplants [3, 4]. Unfortunately, his study just included the presence and not the width of the keratinized tissue, which is a limitation since the width seems to correlate with the occurrence of deeper pocket depths [43]. The presence of keratinized gingiva around the implants did not have a significant influence on rHb or blood flow values, which were the ones we found altered in peri‐implantitis, but there were significant differences in SO_2_ values in free gingiva around the implants. This finding could be explained with a greater density of vessels in non‐keratinized gingiva compared with keratinized gingiva, however, this seems not to be a relevant factor in the onset of peri‐implant disease.

The main limitation of this study is that it includes a clinical cohort, which is on the one hand very vulnerable to peri‐implant disease, but on the other hand brings a lot of cofactors like altered soft tissue, different bone quality, and often compromise‐prone prosthetic restorations.

To minimize the influence of these parameters, we conducted a linear mixed regression model to adjust for the type of microvascular transplant, implant material, keratinized gingiva, and implant type. Including the patient as the random variable, the difference in flow was no longer significant (*p* = 0.400), which shows that there are interindividual differences, that seem to influence the blood flow value as well. This is a limitation in distinguishing clearer boundaries between the groups. A greater and more homogenous study collective or split‐mouth studies with patients who have as well healthy and as also implants affected by peri‐implant disease could be able to address this issue and to provide clearer differences in perfusion parameters. Nevertheless, this does not affect the results of the ROC analysis, which showed with a 100% sensitivity and 60% specificity that blood flow values above 46.5 AU indicate peri‐implantitis. For future studies, it would be interesting to examine the progression of perfusion parameters during recall phases to improve understanding of the correlation between changes in these measurements and shifts in the clinical context.

## 5 Conclusion

Implants in microvascular bone transplants are more vulnerable to peri‐implant disease than implants in native bone [10, 11, 12, 27, 28]. Lack of keratinized tissue, plaque control, and the type of restoration and implant should be addressed, as these are important parameters in the onset of peri‐implant mucositis and peri‐implantitis. An early diagnosis of peri‐implant disease is important to return to implant health and ensure a high implant survival rate.

In this study, implants with peri‐implantitis showed a significantly higher rHb and blood flow value, which shows that peri‐implant perfusion is affected and altered by the inflammation. However, patient‐specific parameters also appear to influence blood flow values, which can obscure the clear distinction between healthy and pathological peri‐implant blood flow. Likewise, BOP around implants can also be prone to false results. Nevertheless, implants with blood flow values > 46.5 AU should be carefully monitored in a regular recall interval or treated when bone loss is also visible radiographically.

In summary, LDF‐TS could support clinical diagnosis by providing additional insights into circulation and microvascular perfusion of the adjacent tissue, thereby enhancing our understanding of peri‐implant inflammation.

As there is still a lack of data about peri‐implant perfusion measurements in healthy and affected implants, future studies with a different patient cohort, such as implants placed in native bone or avascular bone grafts should analyze the relationship between altered perfusion values and signs of peri‐implant disease. Furthermore, the influence of the peri‐implant soft tissue, smoking, or radiation on peri‐implant perfusion is not fully understood and further research is required to get more insights into the vascular degeneration which unfolds around implants with peri‐implant disease.

## Author Contributions

Conceptualization, M.S.K., and A.M.; methodology, M.S.K., and A.M.; validation, M.S.K.; formal analysis, M.S.K., M.O., A.B., N.V., M.H., and K.K.; investigation, M.S.K.; resources, F.H.; data curation, M.S.K., M.H., and F.H.; writing – original draft preparation, M.S.K.; writing – review and editing, N.V., A.B., K.K., M.H., F.H., M.O., and A.M.; visualization, M.S.K.; supervision, A.M.; project administration, M.S.K., A.M., and F.H. All authors have read and agreed to the published version of the manuscript.

## Ethics Statement

All procedures performed in this study involving human participants were in accordance with the ethical standards of the institutional and/or national research committee and with the 1964 Helsinki Declaration and its later amendments or comparable ethical standards. The study was approved by the institutional Clinical Research Ethics Committee (Decision Number 23–108) and registered by the German Clinical Trials Register (File Number DRKS00034432, registered on June 7, 2024).

## Consent

Patients signed informed consent regarding publishing their data and photographs.

## Conflicts of Interest

The authors declare no conflicts of interest.

## Data Availability

The data that support the findings of this study are available on request from the corresponding author. The data are not publicly available due to privacy or ethical restrictions.
